# Microglial K^+^ Channel Expression in Young Adult and Aged Mice

**DOI:** 10.1002/glia.22776

**Published:** 2014-12-03

**Authors:** Tom Schilling, Claudia Eder

**Affiliations:** Institute for Infection and Immunity, St. George's, University of London; Cranmer TerraceLondon, SW17 0RE, United Kingdom

**Keywords:** brain macrophages, aging, development, ion channels, potassium channels

## Abstract

The K^+^ channel expression pattern of microglia strongly depends on the cells' microenvironment and has been recognized as a sensitive marker of the cells' functional state. While numerous studies have been performed on microglia *in vitro*, our knowledge about microglial K^+^ channels and their regulation *in vivo* is limited. Here, we have investigated K^+^ currents of microglia in striatum, neocortex and entorhinal cortex of young adult and aged mice. Although almost all microglial cells exhibited inward rectifier K^+^ currents upon membrane hyperpolarization, their mean current density was significantly enhanced in aged mice compared with that determined in young adult mice. Some microglial cells additionally exhibited outward rectifier K^+^ currents in response to depolarizing voltage pulses. In aged mice, microglial outward rectifier K^+^ current density was significantly larger than in young adult mice due to the increased number of aged microglial cells expressing these channels. Aged dystrophic microglia exhibited outward rectifier K^+^ currents more frequently than aged ramified microglia. The majority of microglial cells expressed functional BK-type, but not IK- or SK-type, Ca^2+^-activated K^+^ channels, while no differences were found in their expression levels between microglia of young adult and aged mice. Neither microglial K^+^ channel pattern nor K^+^ channel expression levels differed markedly between the three brain regions investigated. It is concluded that age-related changes in microglial phenotype are accompanied by changes in the expression of microglial voltage-activated, but not Ca^2+^-activated, K^+^ channels.

## Introduction

Microglial cells undergo a variety of age-related changes in morphology, phenotype and functions (Mosher and Wyss-Coray, 2014; Wong, 2013). A large proportion of microglial cells in the aged brain is characterized by decreased ramification and/or a dystrophic morphology (Streit and Xue, 2013,2014), by a reduction in microglial process motility and cellular migration (Damani et al., 2011; Hefendehl et al., 2014), by changes in basal and stimulated cytokine release (Njie et al., 2012; Sierra et al., 2007) and by impaired phagocytic activity (Mosher and Wyss-Coray, 2014; Njie et al., 2012). Although it has been recognized that aged microglial cells are dysfunctional, their role is not completely understood. Aged microglial cells have an impaired capability of producing antiinflammatory substances, resulting in reduced neuroprotection, whereas expression and production of proinflammatory substances have been found to be enhanced. Thus, it has been suggested that aging shifts microglial polarization toward a proinflammatory M1-like phenotype (Mosher and Wyss-Coray, 2014). However, unlike young microglial cells, aged microglial cells are also characterized by reduced capability to adequately respond to inflammatory stimuli (Mosher and Wyss-Coray, 2014; Streit and Xue, 2013).

Ion channels are essential regulators of a variety of microglial functions, including proliferation, shape changes, migration, cytokine release and reactive oxygen species production. The ion channel expression pattern of microglial cells is tightly controlled, while expression of most ion channel types strongly depends on the cells' functional state (Eder, 1998,2005,2010; Kettenmann et al., 2011). *In situ* recordings from activated proinflammatory microglia revealed an upregulation of inward rectifier K^+^ channels, outward rectifier K^+^ channels and BK-type Ca^2+^-activated K^+^ channels (Bordey and Spencer, 2003; Lyons et al., 2000; Menteyne et al., 2009; Schilling and Eder, 2007). This study was performed to determine whether age-dependent changes in microglial phenotype are accompanied by changes in the expression of functional ion channels, in particular in K^+^ channels.

## Materials and Methods

### Preparation of Brain Slices

Brain slices were prepared from young adult (2–3 months) and aged (19–24 months) C57BL6 mice (Harlan Laboratories UK, Bicester, UK). After dislocation of the neck, mice were decapitated and the brain was removed. Tissue blocks containing the occipital, parietal and temporal lobe, including the entorhinal cortex, were mounted on a vibratome (Dosaka EM Co., Kyoto, Japan) in a chamber filled with cooled artificial cerebrospinal fluid (ACSF), containing (in mM): NaCl, 129; KCl, 3; MgSO_4_, 4; NaHCO_3_, 21; NaH_2_PO_4_, 1.25; CaCl_2_, 0.5; and d-glucose, 10. Horizontal slices of 300 μm thickness were made and transferred to a chamber where they were maintained in oxygenated (95% O_2_, 5% CO_2_) ACSF at room temperature. Similarly, coronal slices consisting of striatum and neocortex were prepared by mounting a tissue block of the frontoparietal lobes on a vibratome. This study was performed in accordance with the Animals (Scientific Procedures) Act 1986 under regulations from the Home Office England.

### Visualization and Identification of Microglial Cells

Microglial cells were identified in brain slices using upright BX51WI microscopes (Olympus, Southend on Sea, UK) equipped with a 60× water immersion objective and either a Hamamatsu Orca 03G camera (Till Photonics GmbH, Munich, Germany) or a F-View II camera (Olympus, Southend on Sea, UK). To stain microglia, brain slices were incubated for 20 min at room temperature in oxygenated ACSF containing 10 µg/mL Alexa488-IB_4_ (Isolectin GS-IB_4_ from Griffonia simplicifolia, Alexa Fluor 488 conjugate; Life Technologies, Paisley, UK) as described previously (Schilling and Eder, 2007). For intracellular staining of microglia, 2 µM Alexa647 (Alexa Fluor 647 hydrazide, tris(triethylammonium) salt, Life Technologies, Paisley, UK) were added to the intracellular solution as described previously (Schilling and Eder, 2007). Staining with Alexa dyes does not affect microglial K^+^ channel activity (Schilling and Eder, 2007).

### Electrophysiological Recordings

Passive membrane properties and membrane currents of microglia in brain slices were measured using the whole-cell configuration of the patch-clamp technique as described previously (Schilling and Eder, 2007). An EPC-10 patch-clamp amplifier (HEKA, Lambrecht/Pfalz, Germany) was interfaced to a computer for pulse application and data recording using the program PatchMaster (HEKA). Patch electrodes of 3–5 MΩ were fabricated on a two-stage puller (Narishige PC-10, Tokyo, Japan) from borosilicate glass (Hilgenberg, Malsfeld, Germany). For current recordings, slices were transferred to a recording chamber where they were superfused at a rate of 3 mL/min with extracellular solution containing (in mM): NaCl, 129; KCl, 3; MgSO_4_, 1.8; NaHCO_3_, 21; NaH_2_PO_4_, 1.25; CaCl_2_, 1.6; D-glucose, 10 (oxygenated with 95% O_2_, 5% CO_2_; pH = 7.4). For measurements of input resistance, cell capacitance, resting membrane potential and voltage-activated K^+^ currents, patch electrodes were filled with the following intracellular solution (in mM): KCl, 120; CaCl_2_, 1; MgCl_2_, 2; HEPES, 10; EGTA, 11 and Alexa647, 0.002 (pH = 7.3). To measure Ca^2+^-activated K^+^ currents (and corresponding cell capacitances and resting membrane potentials), the intracellular solution contained (in mM): KCl, 120; BAPTA, 5; CaCl_2_, 4.73; MgCl_2_, 2; HEPES, 10 and Alexa647, 0.002 (pH = 7.3). The free Ca^2+^ concentration of these intracellular solutions was 100 nM and 5 µM, respectively, as calculated using the program WINMAXC (Chris Patton, Stanford University, CA). All recordings were done at a temperature of 23–26°C. Whole-cell currents were filtered at 3 kHz and stored for subsequent analyses. Analyses were performed with the program FitMaster (HEKA, Lambrecht/Pfalz, Germany). Data are presented as mean values ± standard error of the mean (SEM). The numbers of experiments are indicated.

### Determination of Cell Capacitances, Input Resistances, Resting Membrane Potentials, and Current Densities

Patch pipette capacitance, cell capacitance and series resistance were routinely compensated by the EPC-10 amplifier. Cell capacitances (*C*_slow_ values) measured by the amplifier were used to determine the surface area of individual cells assuming a specific membrane capacitance of 1 µF/cm^2^. Resting membrane potentials were determined in the current-clamp mode directly after establishment of the whole-cell configuration, while no holding current was applied. Leak currents were measured by applying a 10 mV voltage pulse from the holding potential and were used to calculate the input resistance. To determine K^+^ current densities of individual cells, K^+^ current peak amplitudes were measured, leak-subtracted and subsequently normalized to the cell surface area.

### Morphometric Quantification

In this study, ramified microglia were defined as cells with distinct processes at least greater than one cell diameter in length (Korotzer and Cotman, 1992), and dystrophic microglia were defined as cells with fragmented soma and reduced number and length of cell processes (Streit et al., 2004). For quantitative evaluation of microglial morphology, we determined cell soma area, soma solidity, the number of cell processes and the ramification index of each individual microglial cell. The ramification index was determined using the formula: RI=cell area/convex area, where “convex area” is the area of a polygonal object that is defined by the cell's most prominent projections (Eder et al., 1999). Soma solidity as a measure for soma fragmentation was defined as the ratio between the area of the soma and its convex hull as described previously (Soltys et al., 2001). All morphometric analyses were performed using the program Image J (NIH, Bethesda, MA).

### Statistics

The statistical significance of differences between experimental groups was evaluated by one-way ANOVA tests using the SPSS program after testing normality of the distribution with a Shapiro-Wilk test. Tukey's test was used for post hoc comparison after confirming homogeneity of variances with Levene's test. Statistical differences between event frequencies were calculated using a two-tailed two-proportion *z*-test. Data were considered to be statistically significant with *P* < 0.05.

## Results

### Morphology of Microglial Cells in Brain Slices From Adult and Aged Mice

**Figure 1:**
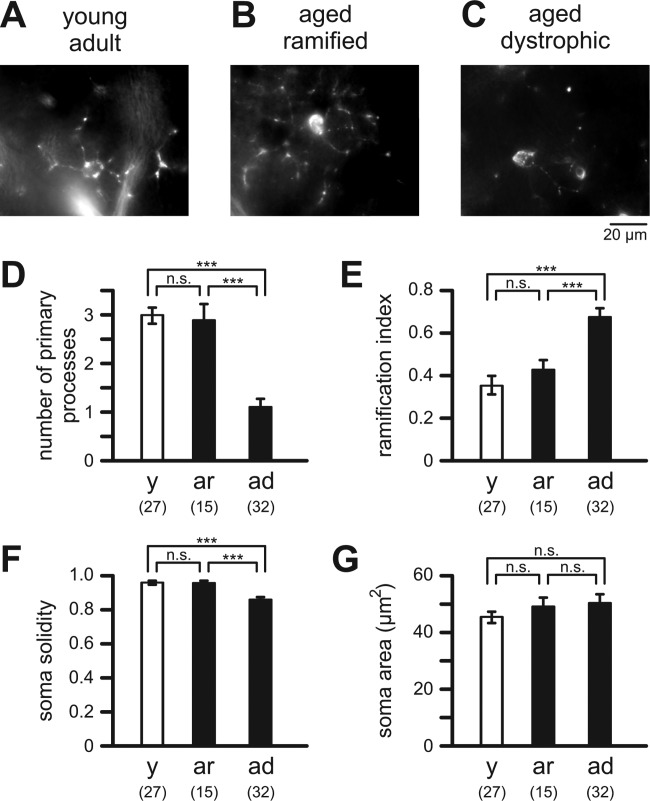
Morphology of microglia in brain slices of young adult (2–3 months) and aged (19–24 months) mice. Isolectin B_4_ fluorescence images of a ramified microglial cell from a young adult mouse (A), a ramified microglial cell from an aged mouse (B), and a dystrophic microglial cell from an aged mouse (C). Quantitative analysis of microglial morphology, i.e., mean number of primary cell processes (D), mean ramification index (E), mean soma solidity (F), and mean soma area (G) of microglial cells in young adult mice (y; open bars), ramified microglial cells in aged mice (ar; closed bars) and dystrophic microglial cells in aged mice (ad; closed bars). Numbers of analyzed cells are indicated. ***, *P* < 0.001; n.s., not significantly different.

Microglial cells in striatum, neocortex, and entorhinal cortex were investigated in slice preparations from young adult (2–3 months) and aged (19–24 months) mice. These brain regions were chosen due to their involvement in age-related neurological disorders. Striatum and neocortex are brain areas heavily affected following ischemic stroke after occlusion of middle cerebral arteries (Durukan and Tatlisumak, 2007; Ekdahl et al., 2009), while the entorhinal cortex is one of the earliest brain areas affected by Alzheimer's disease (Braak and Braak, 1995; Lace et al., 2009). In young adult mice, microglial cells labeled with isolectin B_4_ exhibited a typical ramified morphology characterized by small somata and several branched processes (Fig. 1A). On average, cells had 3.0 ± 0.2 (*n* = 27) primary processes, and a mean ramification index of 0.35 ± 0.03 (*n* = 27) was determined for young adult microglial cells investigated in this electrophysiology study (Fig. 1D,E). As described previously (Streit et al., 2004), microglial morphology in the aged brain was heterogeneous. Some aged microglial cells still exhibited a ramified morphology similar to microglia found in brain slices from young adult mice (Fig. 1B). Neither the average number of primary processes (2.9 ± 0.3, *n* = 15) nor the mean ramification index of ramified microglia in aged mice (0.42 ± 0.05; *n* = 15) differed significantly (*P* = 0.909 and *P* = 0.455, respectively) from those of young adult microglia (see above, Fig. 1D,E). However, a large proportion of microglial cells in brain slices from aged mice exhibited a dystrophic-like morphology characterized by fragmented cell bodies and reduced number of cell processes, which often appeared to be shorter and swollen (Fig. 1C). On average, the number of primary processes of dystrophic microglial cells was 1.1 ± 0.2 (*n* = 32), which was significantly less compared with the number determined for ramified microglia in young adult and aged mice (*P* < 0.001 in both cases; Fig. 1D), and no secondary ramification was observed in most cells investigated. Furthermore, the mean ramification index of dystrophic microglia (0.67 ± 0.03; *n* = 32) was significantly larger than that determined for ramified microglia of both age groups (*P* < 0.001 in both cases; Fig. 1E). Further quantitative analyses revealed that mean soma solidity (Fig. 1F), but not mean soma area (Fig. 1G), of dystrophic microglia in aged mice was significantly smaller than that of ramified microglia in aged and young adult mice (*P* < 0.001 in both cases for soma solidity). Neither soma solidity (*P* = 0.952) nor soma area (*P* = 0.569) were significantly different between ramified microglia of young adult mice (*n* = 27) and ramified microglia (*n* = 15) of aged mice (Fig. 1F,G).

In aged mice, 73% of cells investigated for their electrophysiological properties (*n* = 64) showed morphological signs of dystrophy. The percentage of microglia exhibiting similar dystrophic morphology in young adult mice (4% of 49 cells) was significantly smaller (*P* < 0.001) compared with that in aged mice. During electrophysiological recordings (up to 8 h after slice preparations), morphology of microglial cells in brain slices did not change substantially, i.e., the percentage of dystrophic microglia did not increase with time after slice preparation (data not shown).

### Passive Membrane Properties of Microglia in Brain Slices of Adult and Aged Mice

**Figure 2:**
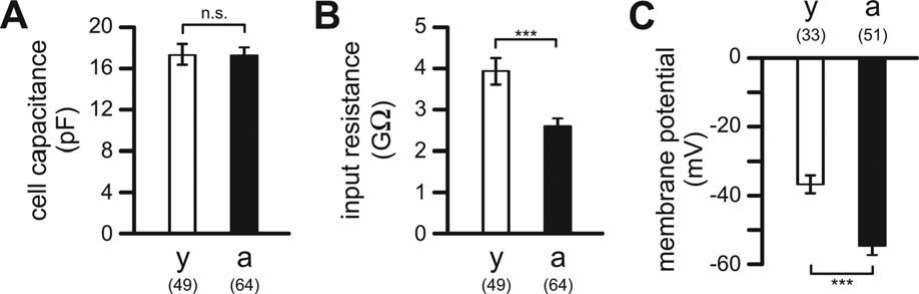
Passive membrane properties of microglial cells in brain slices of young adult (2–3 months) and aged (19–24 months) mice. Mean cell capacitances (A), mean input resistances (B), and mean resting membrane potentials (C) of microglial cells from all brain regions determined in young adult (y; open bars) and aged (a; closed bars) mice. Numbers of analyzed cells are indicated. ***, *P* < 0.001; n.s., not significantly different.

Using the patch clamp technique, passive membrane properties of microglial cells from young adult and aged mice were determined (Fig. 2). Although cell morphology differed substantially between microglial cells of young adult and aged mice, comparison of microglial cell capacitances as a measure of cell surface area did not reveal any significant differences (*P* = 0.928). In young adult mice, microglial cells had a mean cell capacitance of 17.2 ± 1.0 pF (*n* = 49), while a mean cell capacitance of 17.4 ± 0.7 pF (*n* = 64) was determined for microglial cells in aged mice (Fig. 2A). Furthermore, no significant difference (*P* = 0.664) was found between mean cell capacitances of ramified (17.9 ± 1.4 pF; *n* = 17) and dystrophic (17.2 ± 0.8 pF; *n* = 47) microglial cells in aged mice.

In contrast, the mean input resistance of microglial cells (Fig. 2B) was significantly (*P* < 0.001) higher in young adult mice (3.9 ± 0.3 GΩ; *n* = 49) than in aged mice (2.6 ± 0.2 GΩ; *n* = 64), but no significant difference (*P* = 0.181) was found in mean input resistances between ramified (2.2 ± 0.2 GΩ; *n* = 17) and dystrophic (2.7 ± 0.2 GΩ; *n* = 47) microglial cells in the aged brain. Comparison of microglial input resistances between the different brain regions revealed that in young adult mice, the mean input resistance of microglia in neocortex was significantly higher than that of microglia in striatum and entorhinal cortex (*P* < 0.01 in both cases), while in aged mice, no significant differences were found in mean input resistances of microglial cells between the different brain regions (data not shown).

Furthermore, the mean resting membrane potential of microglial cells differed substantially between young adult and aged mice (Fig. 2C). It was significantly (*P* < 0.001) more positive in microglia of young adult mice (−37.1 ± 2.7 mV; *n* = 33) than in microglia of aged mice (−54.3 ± 2.7 mV; *n* = 51). In the aged brain, ramified (−57.2 ± 5.6 mV; *n* = 14) and dystrophic (−53.2 ± 3.1 mV; *n* = 37) microglial cells had almost identical resting membrane potentials (*P* = 0.523). Within each age group, resting membrane potentials of microglial cells from striatum, neocortex and entorhinal cortex did not differ significantly (data not shown).

### Microglial Inward Rectifier K^+^ Currents in Young Adult and Aged Mice

**Figure 3:**
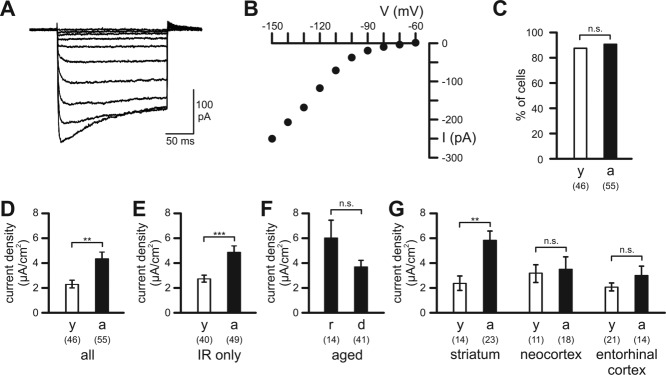
Inward rectifier K^+^ currents of microglial cells. (A) Example of an inward rectifier K^+^ current elicited by 200 ms lasting voltage pulses to potentials between −70 and −150 mV. Holding potential was −60 mV. (B) Current–voltage relationship for the currents shown in (A). (C) Percentage of cells exhibiting inward rectifier K^+^ currents in response to hyperpolarizing potentials. (D) Current densities of inward rectifier K^+^ currents of all microglial cells investigated (all). (E) Current densities of inward rectifier K^+^ currents of only those microglial cells, which expressed the channels (IR only). (F) Current densities of inward rectifier K^+^ currents of all ramified (r) and dystrophic (d) microglial cells in aged mice (aged). (G) Current densities of inward rectifier K^+^ currents of microglia in striatum, neocortex and entorhinal cortex. Open bars: young adult mice (y); closed bars: aged mice (a). Numbers of analyzed cells are indicated. ***, *P* < 0.001; **, *P* < 0.01; n.s., not significantly different.

Microglial K^+^ currents were studied in the whole-cell configuration of the patch clamp technique. To investigate inward rectifier K^+^ currents, microglial cells were clamped at a holding potential of −60 mV, and 200 ms-lasting voltage pulses to potentials between −70 and −150 mV were applied every 10 s. Figure 3 shows a typical example of inward rectifier K^+^ currents (Fig. 3A) and its corresponding current-voltage relationship (Fig. 3B) recorded in a ramified microglial cell within the neocortex of an aged mouse. In young adult mice, the majority (87%; *n* = 46; Fig. 3C) of cells exhibited inward rectifier K^+^ currents in response to hyperpolarizing potentials, and no substantial differences were found between the different brain regions (10 of the 14 cells in striatum, 10 of the 11 cells in neocortex and 20 of the 21 cells in entorhinal cortex). In aged microglia, inward rectifier currents were detected in 89% of microglial cells (*n* = 55; Fig. 3C) with no apparent differences between striatum (23 of the 23 cells), neocortex (14 of the 18 cells), and entorhinal cortex (12 of the 14 cells). Although the percentage of cells exhibiting inward rectifier K^+^ currents did not differ significantly between the two age groups (*P* = 0.741; Fig. 3C), analysis of inward rectifier K^+^ current densities determined at a test potential of −150 mV revealed substantial differences between microglia from young adult and aged mice (Fig. 3D). The mean inward rectifier K^+^ current density of microglia in aged mice (4.3 ± 0.5 µA/cm^2^; *n* = 55), was significantly larger (*P* < 0.01) than that of young adult microglial cells (2.4 ± 0.3 µA/cm^2^; *n* = 46). Similarly, mean inward rectifier K^+^ current density of only those microglial cells expressing the channels was significantly larger (*P* < 0.001) in aged mice (4.88 ± 0.51 µA/cm^2^; *n* = 49) than in young adult mice (2.77 ± 0.28 µA/cm^2^; *n* = 40) (Fig. 3E). In aged mice, ramified and dystrophic microglial cells differed neither in the number of cells expressing inward rectifier K^+^ currents (92.8% of 14 ramified cells, 87.8% of 41 dystrophic cells; *P* = 0.603) nor in the cells' mean inward rectifier K^+^ current density (*P* = 0.103; Fig. 3F). Differences in inward rectifier K^+^ current density between young adult and aged microglia appear to be due to striatal microglia of aged mice, which had a significantly larger mean inward rectifier K^+^ current density than those of young adult mice (*P* < 0.01), while no differences were found between young adult and aged microglial cells in the other two brain regions (Fig. 3G). Although the mean inward rectifier K^+^ current density of striatal microglial cells was significantly increased in aged mice compared with young mice, none of the differences in microglial mean inward rectifier K^+^ current densities between different brain regions reached significance within the two age groups.

### Microglial Outward Rectifier K^+^ Currents in Young Adult and Aged Mice

**Figure 4:**
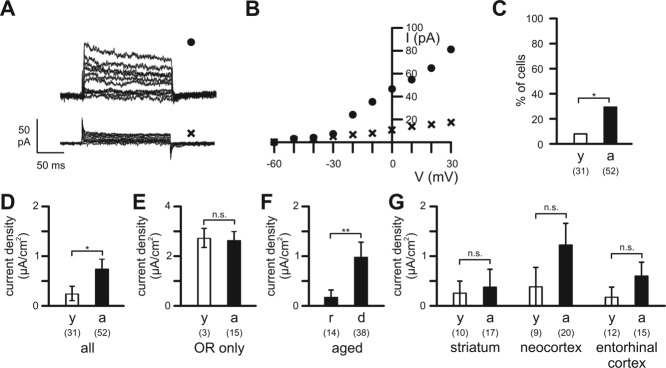
Outward rectifier K^+^ currents of microglial cells. (A) Current recordings in a dystrophic microglial cell of an aged mouse (upper trace, •) and a ramified microglial cell of a young adult mouse (lower trace, x) in response to 200 ms lasting voltage pulses from the holding potential of −60 mV to potentials between −50 and +30 mV. (B) Peak amplitudes of the currents shown in (A) were plotted as a function of membrane voltage. (C) Percentage of cells exhibiting outward rectifier K^+^ currents in young and aged mice. (D) Current densities of outward rectifier K^+^ currents of all microglial cells investigated in young and aged mice (all). (E) Current densities of outward rectifier K^+^ currents of only those microglial cells, which expressed the channels (OR only). (F) Current densities of outward rectifier K^+^ currents detected in ramified (r) and dystrophic (d) microglia of aged mice (aged). (G) Current densities of microglial outward rectifier K^+^ currents from young and aged mice in striatum, neocortex and entorhinal cortex. Open bars: young adult mice (y); closed bars: aged mice (a). Numbers of analyzed cells are indicated. **, *P* < 0.01; *, *P* < 0.05; n.s., not significantly different.

Next, we investigated outward rectifier K^+^ currents in microglia of the different brain regions in young adult and aged mice. In order to elicit outward rectifier K^+^ currents, cells were held at −60 mV and 200 ms-lasting depolarizing voltage steps were applied to potentials between −50 and +30 mV every 10 s. Some microglial cells exhibited outward rectifier K^+^ currents, while others did not show any voltage-activated outward current as shown by their linear current–voltage relationship (Fig. 4A,B). As described before (Eder, 1998; Kettenmann et al., 2011), microglial voltage-gated outward rectifier K^+^ currents activated at potentials more positive than −40 mV and showed time-dependent activation and inactivation. In young adult mice, only a small number of microglial cells (1 of the 10 cells in striatum, 1 of the 9 cells in neocortex, 1 of the 12 cells in entorhinal cortex) exhibited an outward rectifier K^+^ current of small amplitude. The proportion of microglial cells (Fig. 4C) exhibiting outward rectifier K^+^ currents was significantly larger (*P* < 0.05) in aged mice (29%; *n* = 52) than in young adult mice (10%; *n* = 31). The age-dependent increase in outward rectifier K^+^ channel expression was observed in all brain regions investigated (3 of the 17 cells in striatum, 8 of the 20 cells in neocortex, and 4 of the 15 cells in entorhinal cortex of aged mice). Accordingly, mean outward rectifier K^+^ current densities (determined at +30 mV; Fig. 4D) of microglial cells were significantly larger (*P* < 0.05) in aged mice (0.76 ± 0.19 µA/cm^2^; *n* = 52) than in young adult mice (0.26 ± 0.15 µA/cm^2^; *n* = 31). However, in contrast to the upregulated inward rectifier K^+^ channel expression in individual microglial cells of aged mice, expression levels of outward rectifier K^+^ channels in microglial cells expressing these channels did not increase with age. Mean current densities of cells expressing outward rectifier K^+^ channels were 2.7 ± 0.38 µA/cm^2^ (*n* = 3) and 2.63 ± 0.34 µA/cm^2^ (*n* = 15) in young adult and aged mice, respectively, and did not differ significantly (*P* = 0.911; Fig. 4E).

In aged mice, outward rectifier K^+^ currents were found more frequently (*P* < 0.05) in microglial cells exhibiting dystrophic morphology (37%; *n* = 38) than in microglia exhibiting ramified morphology (7%; *n* = 14). In agreement with this observation, mean outward rectifier K^+^ current density of dystrophic microglial cells (0.98 ± 0.25 µA/cm^2^; *n* = 38) was significantly larger (*P* < 0.01) than that of ramified microglia (0.15 ± 0.15 µA/cm^2^; *n* = 14) of aged mice (Fig. 4F). In both age groups, comparison of mean outward rectifier K^+^ current densities of microglial cells between the different brain regions did not reveal any significant differences (Fig. 4G).

### Microglial Ca^2^^+^-Activated K^+^ Currents in Young Adult and Aged Mice

To measure Ca^2+^-activated K^+^ currents, microglial cells were perfused with an internal solution containing 5 µM free Ca^2+^. To inactivate voltage-dependent outward rectifier K^+^ channels, cells were clamped to a holding potential of −20 mV. Voltage pulses to potentials between −10 and +80 mV were applied for 400 ms in 20 mV steps every 10 s. In contrast to cultured microglia (Eder et al., 1997; Khanna et al., 2001), voltage-independent IK-type or SK-type Ca^2+^-activated K^+^ currents could not be detected in microglial cells of any brain region. Perfusion of microglial cells with intracellular solutions containing free Ca^2+^ concentration ([Ca^2+^]) of either 100 nM or 5 µM did not significantly change the mean voltage-independent conductance of microglial cells determined at potentials between −80 and −40 mV in young adult (*n* = 31 cells at 100 nM [Ca^2+^] and *n* = 32 cells at 5 µM [Ca^2+^], *P* = 0.935) or aged mice (*n* = 34 cells at 100 nM [Ca^2+^] and *n* = 31 cells at 5 µM [Ca^2+^], *P* = 0.648). Furthermore, mean membrane potentials of microglial cells were not significantly different between cells perfused with solutions of either 100 nM or 5 µM [Ca^2+^] (*n* = 31 cells at 100 nM [Ca^2+^] and *n* = 32 cells at 5 µM [Ca^2+^], *P* = 0.983 in young adult mice; *n* = 34 cells at 100 nM [Ca^2+^] and *n* = 31 cells at 5 µM [Ca^2+^], *P* = 0.377 in aged mice), indicating the absence of voltage-independent Ca^2+^-activated K^+^ currents in both age groups.

**Figure 5:**
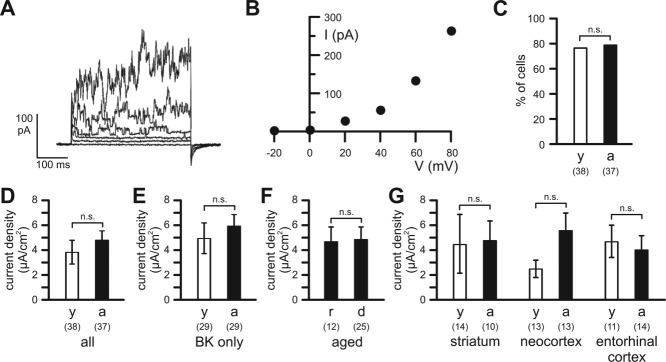
Ca^2+^-activated K^+^ currents of microglial cells. (A) Ca^2+^-activated K^+^ currents elicited by 400 ms lasting voltage pulses from the holding potential of −20 mV to potentials between 0 and +80 mV of a microglial cell in a young adult mouse. (B) Current–voltage relationship for the currents shown in (A). (C) Percentage of cells exhibiting Ca^2+^-activated K^+^ currents in young and aged mice. (D) Current densities of BK-type Ca^2+^-activated K^+^ currents of all microglial cells investigated in young and aged mice (all). (E) Current densities of BK-type Ca^2+^-activated K^+^ currents of only those microglial cells, which expressed the channels (BK only). (F) Current densities of Ca^2+^-activated K^+^ currents of ramified (r) and dystrophic (d) microglia of aged mice (aged). (F) Current densities of Ca^2+^-activated K^+^ currents of microglia in striatum, neocortex and entorhinal cortex. Open bars: young adult mice (y); closed bars: aged mice (a). Numbers of analyzed cells are indicated. n.s., not significantly different.

However, the majority of microglial cells (76% of 38 cells in young adult mice; 78% of 37 cells in aged mice; *P* = 0.833) exhibited BK-type Ca^2+^-activated K^+^ currents at potentials more positive than 0 mV (Fig. 5A–C). No significant differences (*P* = 0.544) were found in the activation threshold of Ca^2+^-activated K^+^ currents between microglial cells in young adult (17 ± 3 mV; *n* = 29) and aged (20 ± 3 mV; *n* = 29) mice. In contrast to voltage-gated K^+^ channel expression of microglia, Ca^2+^-activated K^+^ channel expression did not change in microglia with age. The mean Ca^2+^-activated K^+^ current density (determined at +60 mV) of microglial cells in young adult mice (3.8 ± 1.0 µA/cm^2^; *n* = 38) did not differ significantly (*P* = 0.274) from that of microglia in aged mice (4.8 ± 0.8 µA/cm^2^; *n* = 37; Fig. 5D). Correspondingly, no difference (*P* = 0.462) was found in the mean Ca^2+^-activated K^+^ current densities of microglial cells expressing the channel between young adult (4.99 ± 1.21 µA/cm^2^; *n* = 29) and aged mice (6.08 ± 0.81 µA/cm^2^; *n* = 29) (Fig. 5E). Furthermore, no significant differences (*P* = 0.927) were found in mean Ca^2+^-activated K^+^ current densities between ramified (4.7 ± 1.1 µA/cm^2^, *n* = 12) and dystrophic (4.8 ± 1.0 µA/cm^2^, *n* = 25) microglial cells in aged mice (Fig. 5F), and Ca^2+^-activated K^+^ channel expression did not differ between brain regions within the two age groups (Fig. 5G).

## Discussion

In summary, results of our experiments suggest that passive membrane properties and K^+^ channel expression of microglial cells undergo substantial changes upon aging of the brain. In comparison with microglia of young adult mice, microglial cells of aged mice are characterized by more negative resting membrane potentials, decreased input resistances and upregulated expression of inward rectifier and outward rectifier K^+^ channels.

It has recently been reported that K^+^ channel expression of microglial cells in brain slices from juvenile mice (P5-P9) differs to some extent from that of cells in adult mice (Arnoux et al., 2013,2014; Boucsein et al., 2003; Menteyne et al., 2009; Schilling and Eder, 2007). Here, we have extended these studies by additionally investigating K^+^ currents in microglia of aged mice. Intriguingly, both inward rectifier and outward rectifier K^+^ channel expression is strongly age-dependent, whereas expression of Ca^2+^-activated K^+^ channels remains unchanged in murine microglia upon brain aging. Although current densities of both inward rectifier and outward rectifier K^+^ channels were found to be larger in microglia of aged mice than in microglia of young adult mice, different mechanisms are responsible for these results. The increased inward rectifier K^+^ current density can be explained by enhanced expression of these channels in individual cells, as the percentage of microglial cells expressing these channels does not differ between microglia of young adult and aged mice. In contrast, the percentage of microglial cells expressing outward rectifier K^+^ channels increased with age, whereas the mean outward rectifier current K^+^ density of individual cells in young adult and aged mice remained unchanged. Thus, the increased mean outward rectifier K^+^ current density of aged microglia is solely due to the increased number of cells expressing these channels.

Whereas inward rectifier K^+^ channels are constitutively expressed by murine microglia, outward rectifier K^+^ channels are expressed in the majority of resting microglial cells in juvenile mice (Arnoux et al., 2013,2014; Menteyne et al., 2009;Schilling and Eder, 2007), while their expression is strongly reduced in microglia of young adult mice (Boucsein et al., 2003), but upregulated again in a percentage of microglial cells in aged mice (present study). *In vitro* and *in vivo* studies have suggested that outward rectifier K^+^ channels are a hallmark of activated microglial cells (Nörenberg et al., 1992,1994; reviewed in Eder, 1998,2010; Kettenmann et al., 2011). Thus, it is possible that in the developing brain of juvenile mice, outward rectifier K^+^ channels are required to keep microglial cells at a certain activation state, which is optimal for phagocytosis of apoptotic neurons and cell debris during brain development. In the aged brain, we observed an increase in the number of cells exhibiting outward rectifier K^+^ currents mainly in dystrophic cells. As dystrophic/senescent microglial cells appear to be at a primed, partially activated functional state, i.e., are capable of producing proinflammatory cytokines and reactive oxygen species, it is possible that outward rectifier K^+^ channels are required to keep the cells' functional state. A role of Kv1.3 outward rectifier K^+^ channels in priming of microglial ROS production has recently been suggested (Schilling and Eder, 2011). Further studies are required to test whether upregulated K^+^ channels play an active role in the production and release of proinflammatory substances from aged microglial cells. In contrast, Kv1.3 channel upregulation in the plasma membrane has been related to cell apoptosis (Szabò et al., 2010). Thus, it is also possible that upregulation of outward rectifier K^+^ channels is a signature of aged microglial cells undergoing apoptotic cell death.

To date, no study has addressed the functional role of inward rectifier K^+^ channels in microglia *in vivo*, although it has been found previously that these channels are upregulated in activated microglia *in situ* and *in vivo* (Lyons et al., 2000; Schilling and Eder, 2007), suggesting a specific function in regulating microglial activation mechanisms. Further studies are required to elucidate the role of inward rectifier K^+^ channels in activated and aged microglia *in vivo*. In cultured microglia, inward rectifier K^+^ channels have been found to set the cells' negative membrane potential (Eder, 1998; Kettenmann et al., 2011). Thus, the upregulated expression of inward rectifier K^+^ channels in aged microglia may explain why microglia in aged mice have a significantly more negative resting membrane potential than microglia in young adult mice.

BK-type Ca^2+^-activated K^+^ channels have been identified in cultured bovine and human microglia (McLarnon et al., 1995,1997), but have not been found in cultured murine microglia. However, in brain slices from juvenile mice, activated, but not resting, microglial cells expressed Ca^2+^-activated K^+^ currents (Schilling and Eder, 2007). In contrast to juvenile resting microglia, Ca^2+^-activated K^+^ channel expression was upregulated in microglia of brain slices from healthy adult and aged mice. The two opposite effects, namely upregulation of Ca^2+^-activated K^+^ channels and simultaneous downregulation of voltage-gated outward rectifier K^+^ channels of microglia in the young adult brain, suggest that different K^+^ channel types are responsible for different microglial functions in juvenile and adult mice. In the healthy adult brain, fully differentiated microglial cells mainly have a surveying function. By extending and retracting their processes, microglial cells constantly survey microenvironment and functional state of surrounding neurons (Wake et al., 2013). What might be the function of Ca^2+^-activated K^+^ channels in adult and aged microglia? We suggest the following scenario: local changes in intracellular Ca^2+^ concentration within microglial processes are required for cytoskeletal reorganization during process motility, while increases in the intracellular Ca^2+^ concentration are sufficient to activate Ca^2+^–dependent K^+^ channels. K^+^ efflux through these channels reduces the osmolarity within cell processes, which subsequently would cause water efflux and process retraction. In addition to their proposed role in process motility, Ca^2+^-activated K^+^ channels may be required for controlling and regulating other Ca^2+^-dependent processes, such as the release of neurotrophic substances.

In contrast to age-dependent changes, K^+^ channel expression of microglial cells was found to be almost identical in different brain regions, i.e., in striatum, neocortex and entorhinal cortex from adult and aged mice. These data suggest that in the brain of healthy adult and aged mice, microenvironment and functional state of microglial cells do not differ substantially between different brain regions. However, to date changes in microglial K^+^ channel expression have been detected upon rapid changes in the cells' microenvironment. It is possible that upregulation of certain K^+^ channels reflect immediate/rapid changes in the microenvironment and subsequently in the functional state of microglial cells, rather than persistent differences in microenvironment between individual brain regions. Further studies are required to obtain a better understanding of expression, regulation and functional roles of microglial K^+^ channels *in vivo*.
